# Enhanced activity of an ADAMTS‐13 variant (R568K/F592Y/R660K/Y661F/Y665F) against platelet agglutination *in vitro* and in a murine model of acute ischemic stroke

**DOI:** 10.1111/jth.14275

**Published:** 2018-10-24

**Authors:** K. South, F. Denorme, I. I. Salles‐Crawley, S. F. De Meyer, D. A. Lane

**Affiliations:** ^1^ Centre for Haematology Imperial College London London UK; ^2^ Laboratory for Thrombosis Research KU Leuven Campus Kulak Kortrijk Kortrijk Belgium; ^3^ Present address: Division of Neuroscience University of Manchester Manchester UK

**Keywords:** ADAMTS‐13 protein, conformational activation, hemostasis, stroke, von Willebrand factor

## Abstract

**Essentials**

- ADAMTS13 requires a substrate‐induced conformational change to attain full activity *in vitro*.
- The efficacy of wild type ADAMTS13 in models of thrombosis/stroke may be enhanced by pre‐activation.
- A pre‐activated ADAMTS13 variant exhibits enhanced proteolysis of platelet agglutinates.
- This ADAMTS13 variant is protective in a murine model of stroke at a lower dose than WT ADAMTS13.

**Summary:**

**Background**

ADAMTS‐13 circulates in a closed conformation, only achieving full proteolytic activity against von Willebrand factor (VWF) following a substrate‐induced conformational change. A gain‐of‐function (GoF) ADAMTS‐13 variant (R568K/F592Y/R660K/Y661F/Y665F) is conformationally preactivated.

**Objectives**

To establish how the hyperactivity of GoF ADAMTS‐13 is manifested in experimental models mimicking the occlusive arterial thrombi present in acute ischemic stroke.

**Methods**

The ability of GoF ADAMTS‐13 to dissolve VWF–platelet agglutinates was examined with an assay of ristocetin‐induced platelet agglutination and in parallel‐flow models of arterial thrombosis. A murine model of focal ischemia was used to assess the thrombolytic potential of GoF ADAMTS‐13.

**Results**

Wild‐type (WT) ADAMTS‐13 required conformational activation to attain full activity against VWF‐mediated platelet capture under flow. In this assay, GoF ADAMTS‐13 had an EC_50_ value more than five‐fold lower than that of WT ADAMTS‐13 (0.73 ± 0.21 nm and 3.81 ± 0.97 nm, respectively). The proteolytic activity of GoF ADAMTS‐13 against preformed platelet agglutinates under flow was enhanced more than four‐fold as compared with WT ADAMTS‐13 (EC_50_ values of 2.5 ± 1.1 nm and 10.2 ± 5.6 nm, respectively). In a murine stroke model, GoF ADAMTS‐13 restored cerebral blood flow at a lower dose than WT ADAMTS‐13, and partially retained the ability to recanalize vessels when administration was delayed by 1 h.

**Conclusions**

The limited proteolytic activity of WT ADAMTS‐13 in *in vitro* models of arterial thrombosis suggests an *in vivo* requirement for conformational activation. The enhanced activity of the GoF ADAMTS‐13 variant translates to a more pronounced protective effect in experimental stroke.

## Introduction

Evidence has been accumulating that implicates von Willebrand factor (VWF) as an important factor in the pathophysiology of acute ischemic stroke (AIS) 1. VWF is an adhesive plasma glycoprotein (GP) that is secreted and circulates as large globular multimers 2, 3, 4, 5. In this globular conformation, VWF is quiescent and unable to capture platelets. At sites of vascular damage, VWF is tethered to the vessel wall through its constitutively exposed collagen‐binding site in the A3 domain 6, 7. Under the shear force of flowing blood, the unfolding of its A2 domain 8, 9, 10 induces conformational changes at a macromolecular scale 11, and local conformational changes in the A1 domain expose the previously cryptic platelet GP)1b‐binding site 11. The ability of VWF to capture platelets and to initiate the formation of the platelet plug is dependent on its multimeric size, and is regulated by the plasma metalloprotease ADAMTS‐13 12, 13.

Experimental studies in *VWF* ^−/−^ and *ADAMTS13* ^−/−^ mice have confirmed the importance of the VWF–ADAMTS‐13 axis for the extent of ischemic injury 14, 15, 16, 17. Recombinant ADAMTS‐13 (rADAMTS‐13) has systemic antithrombotic activity in *ADAMTS13* ^−/−^ mice 16. Furthermore, rADAMTS‐13 has been shown to have protective effects in murine stroke models without the risk of intracranial hemorrhage associated with recombinant tissue‐type plasminogen activator (rt‐PA) 18. The VWF content of patient clots has been suggested to be a determining factor in rt‐PA resistance 19. Moreover, a recent study identified VWF‐rich patient clots and demonstrated the efficacy of rADAMTS‐13 against rt‐PA‐resistant, VWF‐rich thrombi in mice 20.

Recently, our understanding of ADAMTS‐13 function has developed with the identification of a substrate‐induced conformational activation mechanism 21, 22, 23, 24. In this model, ADAMTS‐13 circulates in a quiescent conformation, maintained by an autoinhibitory interaction between its N‐terminal spacer domain and its C‐terminal CUB domains 21. This autoinhibition is relieved upon binding of ADAMTS‐13 to the D4‐CK domains of globular VWF, exposing the crucial spacer domain exosite in readiness for VWF A2 domain unfolding. Exposure of ADAMTS‐13 to the VWF D4‐CK domains results in an approximately three‐fold enhancement of its activity. A gain‐of‐function (GoF) ADAMTS‐13 spacer domain variant (R568K/F592Y/R660K/Y661F/Y665F), which was first described by Jian *et al*. 25, is preactivated because of the mutagenic disruption of the autoinhibitory spacer–CUB domain interaction. This results in enhanced activity, even in the absence of VWF D4‐CK. This variant also has the potential for broadened substrate specificity, and there are reports of *in vitro* proteolytic activity against both fibrinogen and plasminogen 26, 27.

We hypothesized that the previously identified, dose‐dependent activity of wild‐type (WT) ADAMTS‐13 in murine models of stroke may be further improved by conformational preactivation. We tested this hypothesis by using an *in vitro* parallel‐flow model of platelet clumping under arterial shear conditions, before comparing the efficacy of WT ADAMTS‐13 with that of GoF ADAMTS‐13 in *in vitro* models of thrombosis and in a murine model of AIS.

## Materials and methods

### Protein expression and purification

Recombinant human ADAMTS‐13 with a C‐terminal Myc/His_6_ tag in pCDNA3.1 28 was used to generate GoF ADAMTS‐13 (R568K/F592Y/R660K/Y661F/Y665F) by sequential site‐directed mutagenesis. For *in vitro* experiments ADAMTS‐13 was expressed in HEK293S stable cell lines and quantified, in conditioned medium, with an in‐house ELISAs as described previously 28, 29. For half‐life determination, ADAMTS‐13 was expressed in either HEK293S or CHO‐K1 stable cell lines, and purified by fast protein liquid chromatography (FPLC) with zinc‐coupled HiTrap chelating columns (GE Healthcare, Chicago, IL, USA). CHO‐expressed ADAMTS‐13, for use in the murine stroke model, was passed over a hydroxyapatite column to remove contaminating proteins, and the purified ADAMTS‐13 was quantified with an in‐house ELISA and dialyzed into 150 mm NaCl, 20 mm histidine, 2% sucrose, and 0.05% Tween‐80 (pH 7.4). The dimeric VWF D4‐CK domain fragment (VWF residues 1874–2813) in the vector pcDNA 3.1/His was transiently expressed in HEK293S, purified by FPLC, and quantified by ELISA as previously described 30.

### Parallel‐flow assays

For parallel‐flow assays, performed in the absence of coagulation, Vena8 Fluoro+ biochips (Cellix, Dublin, Ireland) were coated with 200 μg mL^−1^ collagen type III (Southern Biotech, Birmingham, AL, USA) and blocked with 1% bovine serum albumin (BSA) and 1 mg mL^−1^ glucose in HEPES buffer. Whole donor blood was collected on PPACK (Sigma, Gillingham, UK) and enoxaparin (low molecular weight heparin from Sanofi‐Aventis, Guildford, UK), and treated with 100 nm prostaglandin E1 (PGE1) (Sigma) and 75 mU mL^−1^ apyrase (Sigma), to prevent platelet activation. Platelets were labeled with 10 μm DiOC_6_ (Sigma), and perfused over the collagen surface at a constant shear rate of 1500 s^−1^ (at which rate platelet capture is VWF‐dependent) for 3.5 min. Adhesion of labeled platelets was visualized by fluorescence imaging at 250‐ms intervals with a × 20 objective, and analyzed with slidebook software to determine platelet coverage (%) at 180 s. To determine the effect of ADAMTS‐13 on platelet capture, the assay was also performed in the presence of WT or GoF ADAMTS‐13 at a range of concentrations, and EC_50_ values were determined from dose–response curves with graphpad 7. To assess the effect of ADAMTS‐13 on preformed platelet aggregates, the assay was altered to allow 5 min of perfusion in the absence of rADAMTS‐13, before a further 5 min of perfusion in the presence of rADAMTS‐13 (WT or GoF). For parallel‐flow assays, performed in the presence of coagulation, Vena8 Fluoro+ biochips were coated with 200 μg mL^−1^ collagen type III and 100 pm tissue factor (Sigma) before being blocked with coagulation buffer (1% BSA, 75 mm CaCl_2_ and 37.5 mm MgCl_2_ in HEPES buffer). Whole human blood was collected in 129 mm trisodium citrate (1 : 10 dilution), and platelets were labeled with 10 μm DiOC_6_. Citrated blood was diluted 9 : 1 with coagulation buffer immediately before perfusion over the collagen/tissue factor‐coated surface at 1500 s^−1^ for 3 min. This was repeated three times to provide uninterrupted flow of coagulating blood for a sufficient time to allow the formation of stable pseudothrombi. This was followed by a further 5 min of perfusion with blood, collection on PPACK and enoxaparin, and supplementation with DiOC_6_‐labeled platelets and rADAMTS‐13 (WT or GoF).

### Platelet agglutination assay

Donor blood was collected in 85 mm sodium citrate, 111 mm glucose, and 71 mm citric acid (pH 4.5). Washed platelets were prepared and treated with 100 nm PGE1 (Sigma) and 75 mU mL^−1^ apyrase (Sigma), to prevent platelet activation. Platelets were incubated with 10 μg mL^−1^ VWF (purified from plasma), and agglutination was initiated by the addition of 0.6 mg mL^−1^ ristocetin (Helena Bioscience, Gateshead, UK). Light transmission was recorded with a Chrono‐Log dual channel aggregometer (Kordia BV, Leiden, the Netherlands). After 5 min, once stable agglutination was observed, ADAMTS‐13 (WT or GoF) was administered and light transmission was recorded for a further 30 min.

### FRETS‐VWF73 assay

The FRETS‐VWF73 assays of ADAMTS‐13 activity were performed as described previously 21, 24. Purified VWF D4‐CK (20–60 nm) was added before a 45‐min preincubation at 37°C. Results were normalized to WT ADAMTS‐13 activity.

### Determination of ADAMTS‐13 half‐life in murine plasma

All animal work was performed in compliance with animal ethics guidelines at Imperial College London according to the UK Home Office's Animals (Scientific Procedures) Act 1986, in accordance with the local ethical law and local ethical committees (KU Leuven, Leuven, Belgium; act no. 87–848) and following guidelines for the care and use of laboratory animals. Age‐matched and sex‐matched C57BL/6 littermates, aged 6–8 weeks, were anesthetized with ketamine (75 mg kg^−1^) and medetomidine (1 mg kg^−1^). The injection of ADAMTS‐13 was performed through the retro‐orbital plexus, and blood samples were taken via the same route into 129 mm citrate. Plasma concentrations of ADAMTS‐13 were determined with an in‐house ELISA as previously described 28, 29. To derive *t*_1/2_ values, data were plotted, by use of a one‐phase decay equation, in graphpad 7.

### Murine focal ischemia stroke model

FeCl_3_‐induced occlusion of the middle cerebral artery (MCA) was performed with surgical techniques described previously 20. Regional cerebral blood flow (rCBF) in the MCA territory was determined by laser Doppler flow monitoring, and infarct size was determined 24 h after occlusion of the MCA by staining brain sections with 2% 2,3,5‐triphenyl‐tetrazolium chloride, both as described previously 20.

## Results

### rADAMTS‐13 requires conformational activation for full function in *in vitro* models of platelet clumping

**Figure 1:**
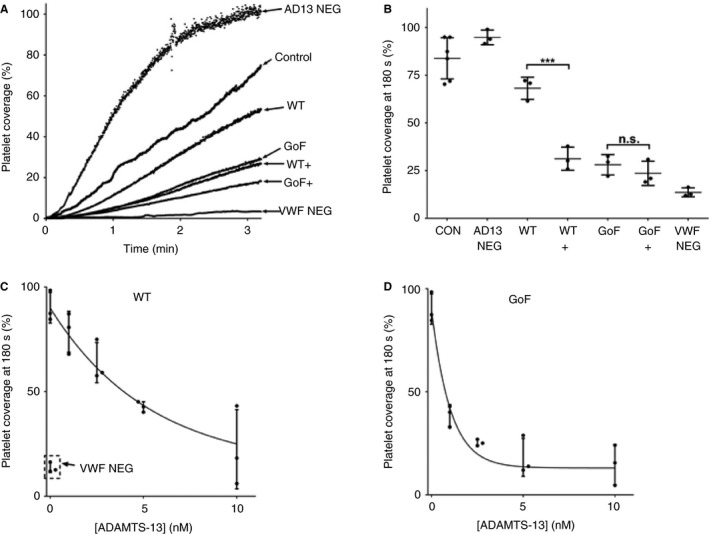
Recombinant wild‐type (WT) ADAMTS‐13 is only partially active in whole blood under flow. (A) Whole human blood, collected on PPACK and clexane, was perfused over a collagen‐coated surface at arterial shear rate, and the von Willebrand factor (VWF)‐mediated capture of labeled platelets (%) was recorded over time (Control). This was repeated with the addition of either 2.5 nm ADAMTS‐13 (WT or gain‐of‐function [GoF]) with or without preincubation with 100 nm VWF D4‐CK (+). As a VWF‐negative control, blood was preincubated with an inhibitory mAb (6B4) to block the VWF–glycoprotein 1b interaction (VWF NEG). As an ADAMTS‐13‐negative control, blood was preincubated with an inhibitory anti‐ADAMTS‐13 mAb (3H9) to inhibit endogenous ADAMTS‐13 (AD13 NEG). The curves are representative of three independent experiments. (B) For each sample, platelet coverage (%) was determined at the end (180 s) of the experiment, and pairwise *t*‐tests were performed between WT and GoF ADAMTS‐13 and their corresponding VWF D4‐CK‐activated samples (***P < 0.005). Results are mean ± standard deviation (SD), *n* = 3–6. (C, D) WT ADAMTS‐13 and the GoF variant were then tested at a range of concentrations in the same assay. For comparison, VWF‐negative controls are included in (C). All results are given as mean ± SD, *n* = 3. CON, control; NS, not significant.

In a previous study of GoF ADAMTS‐13 26, the variant showed increased efficacy in reducing the VWF‐mediated capture of washed plaletets under parallel flow, as compared with WT ADAMTS‐13. This was attributed to the variant's preactivated state and the requirement of WT ADAMTS‐13 for conformational activation. To investigate the dependence of WT ADAMTS‐13 activity on conformational activation by VWF, and the hyperactivity of the GoF variant, in a physiological setting, we performed parallel‐flow assays with whole human blood under arterial shear rates. In these assays, in which platelet capture is VWF‐dependent, the addition of 2.5 nm WT rADAMTS13 elicited only a slight reduction in platelet capture over time (Fig. 1A). When WT ADAMTS‐13 was preincubated with 100 nm VWF D4‐CK prior to being added to the assay, there was a significant decrease (*P* < 0.005) in the platelet coverage recorded at the end of the assay (Fig. 1A,B). The extent of the decrease in platelet capture in the presence of conformationally activated WT ADAMTS‐13 was similar to that observed in the presence of GoF ADAMTS‐13 (Fig. 1A,B). The activity of GoF ADAMTS‐13 was not further enhanced by the addition of VWF D4‐CK. Dose–response curves were generated by titrating both WT and GoF ADAMTS‐13 into the platelet capture assay and determining the platelet coverage after 270 s of blood perfusion (Fig. 1C,D). These plots were used to determine EC_50_ values of 3.81 ± 0.97 nm and 0.73 ± 0.21 nm for WT and GoF ADAMTS‐13, respectively.

### Preactivated GoF ADAMTS‐13 shows enhanced ability to dissolve VWF–platelet agglutinates/aggregates

**Figure 2:**
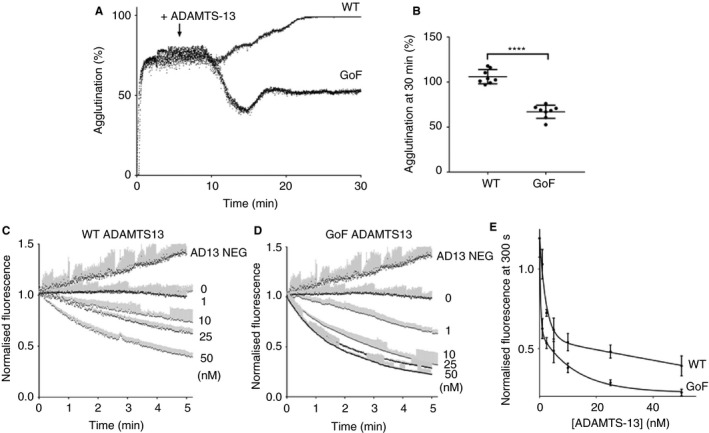
Gain‐of‐function (GoF) ADAMTS‐13 is more effective in the dissolution of preformed von Willebrand factor (VWF)–platelet agglutinates and in the lysis of preformed VWF–platelet aggregates under flow. (A) Agglutination of washed platelets (100 000 μL^−1^) was initiated by the addition of 10 μg mL^−1^ plasma VWF and 0.6 mg mL^−1^ ristocetin. Once maximal agglutination was achieved (5 min), 50 nm ADAMTS‐13 (wild‐type [WT] or GoF variant) was injected, and light transmission was followed for a further 25 min. Curves are representative of *n* = 8. (B) The effect of ADAMTS‐13 on platelet agglutinates was quantified at 30 min. Results are mean ± standard deviation (SD), *n* = 8 (****P < 0.001). (C, D) Whole human blood, collected on PPACK and clexane, containing labeled platelets was perfused over a collagen surface at arterial shear for 5 min to form VWF–platelet aggregates. At the end of this period (*t* = 0 min), the channel was perfused for a further 5 min with blood containing either WT or GoF ADAMTS‐13 (0–50 nm) or blood treated with an inhibitory anti‐ADAMTS‐13 (3H9) mAb (AD13 NEG). Fluorescence was recorded throughout the second period of perfusion, and was normalized to the fluorescence at *t* = 0 min. (E) The normalized fluorescence at *t* = 5 min was determined for each ADAMTS‐13‐treated sample. All results are mean ± SD,* n* = 3.

Having established that WT ADAMTS‐13 requires conformational activation for full activity against VWF in a physiological setting, we hypothesized that this may limit its ability to dissolve preformed VWF–platelet agglutinates, and that the GoF variant would not be limited in this manner. This was first investigated in an agglutination assay with washed platelets. In this assay, ristocetin‐induced VWF unfolding triggers the formation of platelet agglutinates before the addition of ADAMTS‐13. In the presence of WT ADAMTS‐13, the agglutination of platelets continued to increase steadily over the course of 30 min (Fig. 2A,B). In samples treated with GoF ADAMTS‐13, there was initial partial dissolution of platelet agglutinates (Fig. 2A) and a 30–50% decrease in the final agglutination as compared with that in the WT ADAMTS‐13‐treated samples (Fig. 2B; *P* < 0.001).

The ability of ADAMTS‐13 to dissolve preformed VWF–platelet aggregates in whole blood was examined under arterial shear rates, under conditions in which platelet capture was VWF‐dependent (Fig. 1A,B). After 5 min of perfusion of whole blood over a collagen‐coated surface, approximately 60–70% of the surface was covered by labeled platelets. During a second 5‐min period of perfusion with whole blood containing endogenous ADAMTS‐13, this remained stable (Fig. 2C,D). When endogenous ADAMTS‐13 was inhibited (AD13 NEG) by the addition of an inhibitory mAb (3H9), the platelet coverage continued to increase steadily throughout the second period of perfusion (Fig. 2C,D). With titration of WT and GoF ADAMTS‐13 into this assay during the second perfusion period (Fig. 2C,D), EC_50_ values were determined for the dissolution of VWF–platelet aggregates, i.e. 10.2 ± 5.6 nm and 2.5 ± 1.1 nm, respectively (Fig. 2E).

### GoF ADAMTS‐13 reduces the size of *in vitro* preformed pseudothrombi

**Figure 3:**
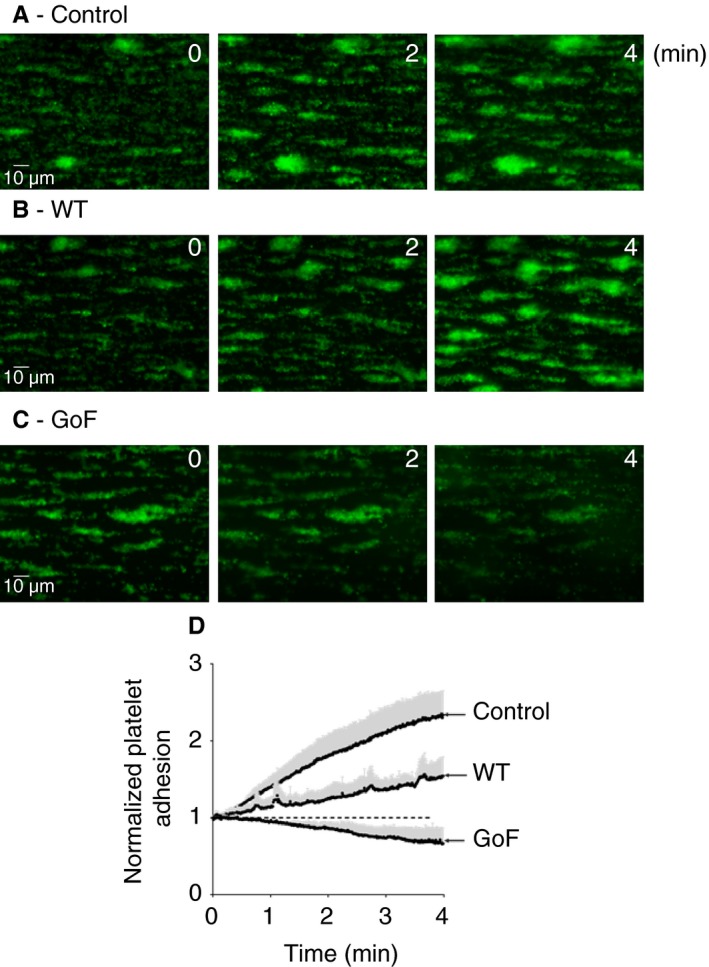
Gain‐of‐function (GoF) ADAMTS‐13 partially dissolves von Willebrand factor (VWF)/platelet‐rich pseudothrombi under flow. (A) Whole human blood, collected on citrate and containing labeled platelets, was recalcified immediately prior to perfusion over a collagen/tissue factor‐coated surface. After 9 min of continuous perfusion, large, VWF/platelet‐rich pseudothrombi (shown previously to also contain fibrin(ogen) 26) were formed. At the end of this period (*t* = 0 min) the channel was perfused for a further 4 min with labeled, recalcified blood, and platelet fluorescence was continuously monitored. (B, C) The assay was repeated with the addition of 5 nm ADAMTS‐13 (wild‐type [WT] or GoF) during the second perfusion period. Images are representative of three independent experiments. (D) Fluorescence readings for each sample were normalized to that at *t* = 0 min, and are expressed as mean ± SD,* n* = 3. [Color figure can be viewed at http://www.wileyonlinelibrary.com]

To examine the ability of WT and GoF ADAMTS‐13 to dissolve preformed pseudothrombi, the parallel‐flow assay with whole blood was adapted to monitor the formation of platelet aggregates in the presence of induced coagulation and fibrin deposition. Unlike the previous parallel‐flow assays performed in the presence of anticoagulants, this assay generated large, stable platelet aggregates (Fig. 3), which have been shown previously to contain fibrin(ogen) 26. When channels containing these pseudothrombi were further perfused with whole blood containing endogenous ADAMTS‐13, there was some remodeling of the pseudothrombi (Fig. 3A), most of which were enlarged, resulting in a steady increase in the DiOC_6_ fluorescence attributable to the ongoing incorporation of platelets into the thrombus (Fig. 3D). In the presence of added WT ADAMTS‐13 (at the normal plasma concentration), there was still enlargement of the pseudothrombi (Fig. 3B); however, the extent of the fluorescence increase was somewhat reduced (Fig. 3D). When treated with GoF ADAMTS‐13, the pseudothrombi underwent extensive remodeling (Fig. 3C), and, at the end of the second perfusion period, the size of the pseudothrombi (as measured by platelet fluorescence) was significantly reduced (*P* < 0.005) as compared with the fluorescence at *t* = 0 (Fig. 3D).

### Characterization of ADAMTS‐13 for *in vivo* administration

**Figure 4:**
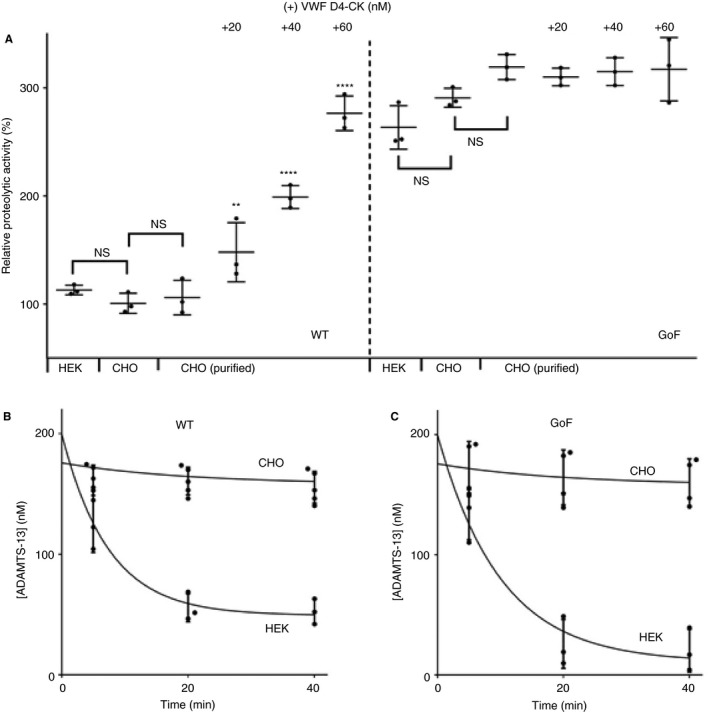
Characterization of recombinant ADAMTS‐13 expressed in CHO cell lines and purified for *in vivo* experiments. (A) The proteolytic activities of ADAMTS‐13 expressed in HEK293S and CHO K1 stable cell lines (in conditioned medium) and purified ADAMTS‐13 expressed in CHO K1 cells were determined with the FRETS‐VWF73 assay. Pairwise *t*‐tests showed no significant difference in the activity of ADAMTS‐13 expressed in the two different cell lines or between purified ADAMTS‐13 and that in conditioned medium. The enhancement of purified ADAMTS‐13 activity by VWF D4‐CK was also determined. The activity of each VWF D4‐CK‐treated sample was compared with the corresponding ADAMTS‐13 activity (**P < 0.01, ****P < 0.001). All results are mean ± standard deviation (SD), *n* = 3. (B, C) Adult wild‐type (WT) C57bl6 mice were injected with purified ADAMTS‐13 (WT or gain‐of‐function [GoF]) expressed in either HEK293S or CHO K1 cell lines. The plasma ADAMTS‐13 antigen level in these mice was determined at 5, 20 and 40 min after injection. Results are mean ± SD,* n* = 3–5. VWF, von Willebrand factor.

For *in vivo* studies, rADAMTS‐13 (WT and GoF) was expressed in CHO‐K1 stable cell lines, which have been shown in previous studies to generate ADAMTS‐13 with a long half‐life in murine experiments. This has been attributed to an altered pattern of glycosylation as compared with ADAMTS‐13 expressed in HEK293 cell lines 14. This material required extensive purification with a combination of techniques, designed to remove protein contaminants without affecting ADAMTS‐13 activity. The results presented so far herein, and in all of our previous studies of GoF ADAMTS‐13 21, 24, 26, have been generated by the use of material expressed in HEK293S cell lines. Characterization of the purified, CHO‐expressed ADAMTS‐13 was therefore required. There was no significant difference in proteolytic activity (against FRETS‐VWF73) of WT ADAMTS‐13 preparations expressed in HEK or CHO cell lines and no significant difference in the activity of CHO‐expressed WT ADAMTS‐13 before and after purification (Fig. 4A). The activity of CHO‐expressed GoF ADAMTS‐13 was enhanced approximately three‐fold as compared with that of WT ADAMTS‐13 (Fig. 4A). This was similar to the activity of the HEK‐expressed GoF ADAMTS‐13, here and in previous studies, and this was not altered upon purification. As expected, the activity of the CHO‐expressed WT ADAMTS‐13, but not that of the GoF variant, was dose‐dependently enhanced by the addition of the VWF D4‐CK domain fragment (Fig. 4A). A comparison of the half‐lives of WT and GoF ADAMTS‐13 confirmed the rapid clearance of HEK‐expressed ADAMTS‐13 from murine plasma (Fig. 4B,C), with *t*_1/2_ values of 10.6 ± 0.45 min and 9.3 ± 0.58 min, respectively. Half‐lives could not be determined for CHO‐expressed ADAMTS‐13, as the plasma concentrations (in mice injected with either WT or GoF ADAMTS‐13) decreased by only 5–10% over the course of the experiment.

### GoF ADAMTS‐13 restores cerebral blood flow after experimental stroke and reduces cerebral ischemia at a lower dose than WT ADAMTS‐13

**Figure 5:**
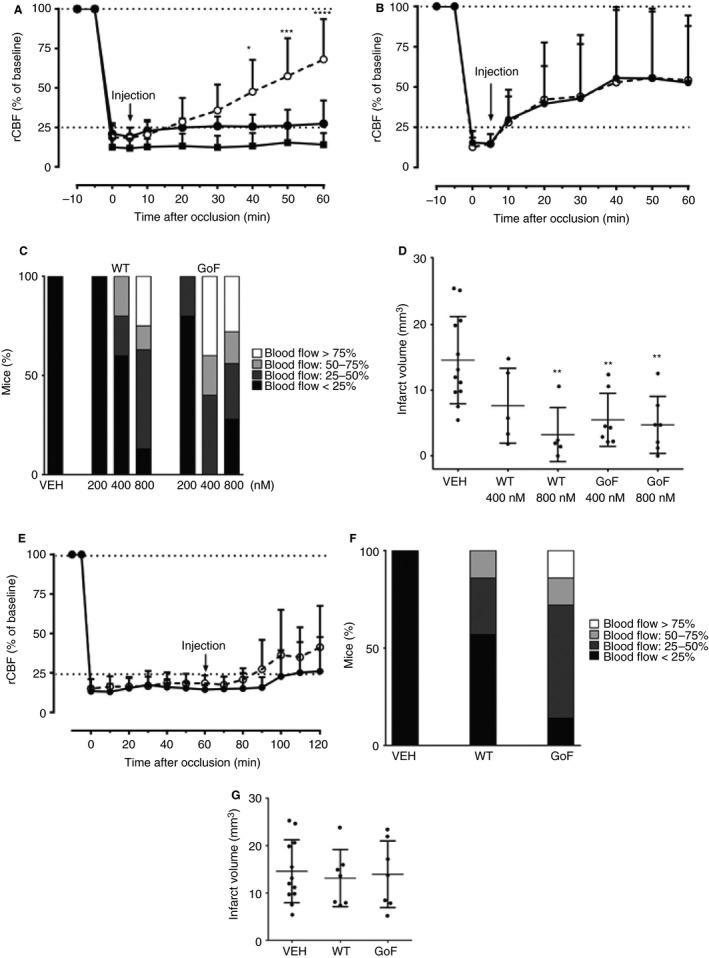
Gain‐of‐function (GoF) ADAMTS‐13 restores cerebral blood flow and reduces infarct size in a murine model of ischemic stroke at a lower dose than wild‐type (WT) ADAMTS‐13. (A, B) Adult WT C57bl6 mice underwent FeCl_3_‐mediated middle cerebral artery occlusion (MCAO), characterized by a reduction in regional cerebral blood flow (rCBF) to below 25% of baseline (determined by laser doppler flowmetry). At 5 min after stable occlusion, mice were injected with either WT ADAMTS‐13 (closed circles), GoF ADAMTS‐13 (open circles) or vehicle only (closed squares, *n* = 12), and rCBF was monitored for a further 60 min. This was performed with an ADAMTS‐13 plasma concentration of either 400 nm (A, *n* = 5) or 800 nm (B, *n* = 7). Results are mean ± standard deviation (SD). Changes in laser Doppler blood flow were analyzed and compared by the use of repeated measures anova. (C) The proportion of mice in which ADAMTS‐13 treatment restored rCBF to > 25%, > 50% or <75%, 60 min after injection. (D) At 24 h after MCAO, mice were killed, and cerebral infarct volume was determined by 2,3,5‐triphenyltetrazolium chloride (Sigma) staining. Stained brain sections were photographed, and infarct areas were analyzed via planimetry with image j software (http://imagej.nih.gov/ij/) by an experimenter who was blinded to treatment conditions. An unpaired Student's *t*‐test or one‐way anova with Dunnett's multiple comparison test was used for statistical comparison of infarct lesions, when applicable (**P < 0.05). Results are mean ± SD. (E) The ability of WT ADAMTS‐13 (800 nm, closed circles) and GoF ADAMTS‐13 (800 nm, open circles) to restore rCBF in the MCAO model was also assessed when administration was delayed by 60 min. Results are mean ± SD,* n* = 7. (F) The proportion of mice in which delayed ADAMTS‐13 treatment restored rCBF to >25%, >50% or <75%, 60 min after injection. (G) No significant reduction in infarct volume was observed in mice treated with delayed ADAMTS‐13 administration (mean ± SD,* n* = 7) as compared with the vehicle group (*n* = 12). VEH, vehicle.

As GoF ADAMTS‐13 shows an increased ability to dissolve VWF/platelet‐rich aggregates *in vitro*, we hypothesized that it may exert a more pronounced protective effect than WT ADAMTS‐13 in an experimental model of stroke. Large, VWF‐rich thrombotic occlusions were generated in the MCAs of C57BL/6 mice. Occlusion was defined by reduction of rCBF to < 25% of baseline (determined by laser Doppler flowmetry) that remained stable for 5 min. In vehicle‐only controls, rCBF remained stable for a period of 60 min, and no spontaneous recanalization, defined as a return of rCBF to > 25% of baseline, was observed (Fig. 5A). Mice were injected with WT or GoF ADAMTS‐13 at a final concentration of 200, 400 or 800 nm, and body weight was used to estimate the total blood volume. There was no restoration of blood flow in any of the mice injected (*n* = 5) with 200 nm WT ADAMTS‐13, and there was minimal restoration of blood flow in only one of the mice injected (*n* = 5) with 200 nm GoF ADAMTS‐13 (Fig. 5C). The mean rCBF of the five mice injected with 400 nm WT ADAMTS‐13 did not significantly increase above the recanalization threshold of 25% (Fig. 5A), with only two mice showing restored blood flow (Fig. 5C). Injection of 400 nm GoF ADAMTS‐13 (*n* = 5) resulted in a mean rCBF that was significantly higher than the recanalization threshold (Fig. 5A), with two mice showing rCBF above 75% (Fig. 5C). At the highest dose tested (800 nm,* n* = 7), both WT and GoF ADAMTS‐13 elicited a rapid and sustained increase in the mean rCBF above the recanalization threshold (Fig. 5B), similar to that seen in previous studies of WT ADAMTS‐13 20. The restoration of blood flow in these mice equated to a significant decrease in infarct volume 24 h after stroke, as compared with the vehicle‐only group (*P* < 0.01). This was true for the groups treated with 400 nm or 800 nm GoF ADAMTS‐13, but only in the 800 nm group for WT ADAMTS‐13 (Fig. 5D).

The ability of GoF ADAMTS‐13 to reduce the size of preformed, stable platelet aggregates *in vitro* suggested that its protective effect in experimental stroke may be sustained even when administration is delayed. Delayed administration of both WT and GoF ADAMTS‐13 at a single dose of 800 nM was evaluated. Although highly variable, and therefore not statistically significant, the injection of GoF ADAMTS‐13 at 60 min after occlusion did result in a small increase in the mean rCBF above the recanalization threshold (Fig. 5E), with six of the seven mice in the group showing some level of blood flow restoration as compared with only two mice in the WT group (Fig. 5F). Despite these results, there was no observable improvement in terms of infarct volume 24 h after stroke (Fig. 5G).

## Discussion

### Under physiological conditions (arterial flow rate in the presence of the plasma concentration of VWF) WT ADAMTS‐13 is not fully active

In previous studies of GoF ADAMTS‐13, first described by Jian *et al*. 25, the hyperactivity of the variant was similar to that of conformationally activated WT ADAMTS‐13 21, 24. Therefore, it is described as being preactivated. In an assay of the VWF‐mediated capture of platelets from plasma‐free blood, performed under flow, the GoF variant showed a similar level of enhanced activity (approximately three‐fold to four‐fold decrease in EC_50_) as that observed in static assays 26. This suggested that, under these conditions, WT ADAMTS‐13 was not conformationally activated. We set out to establish whether or not WT ADAMTS‐13 is conformationally active under conditions that better reflect the physiological setting of arterial thrombus formation, to allow us to establish whether or not preactivated GoF ADAMTS‐13 would be a more potent thrombolytic agent *in vivo*.

In parallel‐flow assays, in which platelet capture is dependent on the unfolding of VWF (present at the normal plasma concentration) by arterial shear, WT ADAMTS‐13 was clearly less active than the GoF variant (Fig. 1A,B). However, preincubation of WT ADAMTS‐13 with 100 nm VWF D4‐CK (2.5 times the plasma concentration of monomeric VWF) results in an activity that is comparable to that of GoF ADAMTS‐13. This strongly suggests that the physiological activity of WT ADAMTS‐13 is, in part, dependent on the availability and/or binding affinity of the D4‐CK domains within plasma VWF. The fact that the activity of GoF ADAMTS‐13 in this assay cannot be further enhanced by VWF D4‐CK is indicative of a preactivated state, as seen in static assays and in previous platelet capture assays 21, 24, 26. This is further supported by the decrease in the EC_50_ determined for the GoF variant as compared with WT ADAMTS‐13 (Fig. 1C,D). The fact that the activity of WT ADAMTS‐13 is dependent, at least in part, on its conformational activation may be an important consideration, as the recombinant protein may be useful as a potential therapy for thrombotic thrombocytopenic purpura 31, 32 and ischemic stroke 20.

### The preactivated state of GoF ADAMTS‐13 increases its ability to dissolve platelet agglutinates/aggregates *in vitro*

WT ADAMTS‐13 requires activation by the D4‐CK domains of VWF to attain full activity against VWF under flow. In a physiological setting, in which VWF binds to collagen and platelets as it is incorporated into the developing platelet plug, reduced access to the activating D4‐CK domains may further limit the efficacy of WT ADAMTS‐13. Therefore, we hypothesized that preactivated GoF ADAMTS‐13 may be better able to proteolyze VWF that is already incorporated into platelet aggregates, thereby reducing the size of preformed thrombi.

We first examined this in an assay of ristocetin‐induced platelet agglutination, allowing the formation of VWF–platelet agglutinates before the addition of ADAMTS‐13. The inability of WT ADAMTS‐13 to dissolve the preformed agglutinates was clearly evident (Fig. 2A,B), in contrast to previous studies in which WT ADAMTS‐13 reduced platelet agglutination 20. This may be the result of differences in ADAMTS‐13 concentration and/or activity between the ADAMTS‐13 used here (prepared in‐house) and the commercial ADAMTS‐13 preparation used previously 20. Comparatively, GoF ADAMTS‐13 showed an ability to reduce the size of the agglutinates, which was evident from the 30–50% reduction in agglutination determined by light transmission measurements (Fig. 2A,B), and from visual inspection of the cuvette contents upon completion. The increased efficacy of GoF ADAMTS‐13 against preformed platelet aggregates was also evident when it was examined under flow, where it showed an EC_50_ value four times lower than that of WT ADAMTS‐13 (Fig. 2C–E). We also employed a more physiologically relevant model in which the complexity of the fibrin‐containing pseudothrombi would be expected to further hinder the conformational activation, and therefore the efficacy, of WT ADAMTS‐13. Indeed, WT ADAMTS‐13, at least at physiological concentration, was unable to reduce the size of the thrombi, and only partially reduced the ongoing incorporation of platelets as compared with the control (Fig. 3A,B,D). The GoF variant, on the other hand, not only prevented ongoing platelet recruitment, but actively remodeled and reduced the size of the pseudothrombi (Fig. 3C,D).

### GoF ADAMTS‐13 shows enhanced thrombolytic activity and is protective in a murine model of AIS

Finally, we examined the efficacy of WT and GoF ADAMTS‐13 in an established murine model of AIS. The formation of occlusive thrombi in this model, achieved through topical administration of FeCl_3_ to the MCA, has been shown previously to be highly dependent on VWF. Therefore, this is a relevant model of rt‐PA‐resistant thrombi, and is useful for demonstrating how targeting VWF can induce recanalization 20. With its preactivated conformation, and enhanced proteolytic activity against VWF (Fig. 4A), it was hypothesized that GoF ADAMTS‐13 would be more effective at dissolving these VWF‐rich thrombi. This was true, to some extent, as GoF ADAMTS‐13 remained protective at a lower dose than WT ADAMTS‐13, in terms of both rCBF restoration (Fig. 5A–C) and infarct volume reduction (Fig. 5D). The fact that GoF ADAMTS‐13 was effective at lower doses than WT ADAMTS‐13 most likely reflects the enhanced activity of the preactivated ADAMTS‐13 variant, which could become useful in the setting of stroke. The enhanced ability of GoF ADAMTS‐13 to reduce the size of established pseudothrombi *in vitro* led us to examine whether or not it would remain effective at restoring blood flow in the AIS model when administration was delayed by 1 h. Again, this was observed to some extent, as a greater proportion of mice treated with GoF ADAMTS‐13 showed partially restored rCBF as compared with those treated with WT ADAMTS‐13 (Fig. 5E,F). However, this was not reflected in infarct volume measurements (Fig. 5G). Previous studies showed that WT ADAMTS‐13 was able to reduce infarct size when administration was delayed by 1 h 20. Although it is difficult to compare the concentrations used in this study, a difference in absolute concentration could explain this difference. The half‐lives of the WT ADAMTS‐13 and GoF ADAMTS‐13 used in these experiments was not determined; however, there was negligible reduction in the plasma concentration of either protein over a similar time period (Fig. 4B,C). Furthermore, the half‐lives of WT and GoF ADAMTS‐13 (expressed in HEK293S cells) in murine plasma were comparable.

In summary, the enhanced activity of preactivated ADAMTS‐13 (as compared with WT ADAMTS‐13) in *in vitro* assays of platelet capture (Fig. 1B) suggests that, at plasma ADAMTS‐13/VWF concentrations, the activity of ADAMTS‐13 can be further increased. The fact that GoF ADAMTS‐13 does not require conformational activation appears to result in increased efficacy in a model of AIS, which may reduce the required dose. Further studies of the therapeutic potential of GoF ADAMTS‐13 in the setting of ischemic stroke, focusing particularly on thromboinflammatory stroke, are required, as is further investigation into the possibility of broadened substrate specificity and the possible impact of this on the variant's safety and efficacy *in vivo*.

## Addendum

K. South, I. I. Salles‐Crawley, and F. Denorme performed research. K. South, F. Denorme, S. F. De Meyer, and D. A. Lane designed research, analyzed data, interpreted data, generated figures, and wrote the paper. All authors read and approved the final version of the manuscript.

## Disclosure of Conflict of Interests

F. Denorme and S. F. De Meyer have a patent WO2016191565 A1 for compositions comprising ADAMTS‐13 for use in methods for the recanalization of occluded blood vessels in an infarction pending. The other authors state that they have no conflict of interest.
